# Health App Use and Its Correlates Among Individuals With and Without Type 2 Diabetes: Nationwide Population-Based Survey

**DOI:** 10.2196/14396

**Published:** 2020-05-20

**Authors:** Lena M Stühmann, Rebecca Paprott, Christin Heidemann, Jens Baumert, Sylvia Hansen, Daniela Zahn, Christa Scheidt-Nave, Paul Gellert

**Affiliations:** 1 Institute for Medical Sociology and Rehabilitation Science Charité – Universitätsmedizin Berlin Berlin Germany; 2 Department of Epidemiology and Health Monitoring Robert Koch Institute Berlin Germany; 3 Cologne Center for Ethics, Rights, Economics, and Social Sciences of Health Cologne Germany; 4 Preventive Cardiology and Medical Prevention, Department of Cardiology University Medical Centre Johannes Gutenberg University Mainz Germany; 5 Federal Center for Health Education (BZgA) Office for National Education and Communication on Diabetes Mellitus Cologne Germany

**Keywords:** mobile app, smartphone, diabetes mellitus, type 2 diabetes, risk factors, health-related behavior, health promotion

## Abstract

**Background:**

Evidence suggests that mobile health app use is beneficial for the prevention and management of type 2 diabetes (T2D) and its associated complications; however, population-based research on specific determinants of health app use in people with and without T2D is scarce.

**Objective:**

This cross-sectional study aimed to provide population-based evidence on rates and determinants of health app use among adults with and without T2D, thereby covering a prevention perspective and a diabetes management perspective, respectively.

**Methods:**

The study population included 2327 adults without a known diabetes diagnosis and 1149 adults with known T2D from a nationwide telephone survey in Germany conducted in 2017. Rates of smartphone ownership and health app use were estimated based on weighted sample proportions. Among smartphone owners, determinants of health app use were identified for both groups separately in multivariable logistic regression models. Sociodemographic factors, diabetes-related factors or indicators, psychological and health-related factors, and physician-provided information were selected as potential determinants.

**Results:**

Among participants without known diabetes, 74.72% (1690/2327) were smartphone owners. Of those, 49.27% (717/1690) used health apps, most often to improve regular physical activity. Among participants with T2D, 42.26% (481/1149) were smartphone owners. Of those, 41.1% (171/481) used health apps, most commonly to target a healthy diet. Among people without known diabetes, determinants significantly (all *P* values <.05) associated with an increased likelihood of health app use compared with their reference group were as follows: younger and middle age of 18 to 44 or 45 to 64 years (odds ratios [ORs] 3.89; *P*<.001 and 1.76; *P*=.004, respectively), overweight or obesity (ORs 1.58; *P*<.001 and 2.07; *P*<.001, respectively), hypertension diagnosis (OR 1.31; *P*=.045), former or current smoking (ORs 1.51; *P*=.002 and 1.58; *P*<.001, respectively), perceiving health as very good (OR 2.21; *P*<.001), other chronic diseases (OR 1.48; *P*=.002), and having received health advice from a physician (OR 1.48; *P*<.001). A slight or high perceived diabetes risk (ORs 0.78; *P*=.04 and 0.23; *P*<.001, respectively) was significantly associated with a decreased likelihood of health app use. Among people with T2D, younger and middle age (18-64 years; OR 1.84; *P*=.007), female gender (OR 1.61; *P*=.02), and using a glucose sensor in addition or instead of a glucose meter (OR 2.74; *P*=.04) were significantly positively associated with health app use.

**Conclusions:**

In terms of T2D prevention, age, diabetes-related risk factors, psychological and health-related factors, and medical health advice may inform app development for specific target groups. In addition, health professionals may encourage health app use when giving advice on health behaviors. Concerning T2D management, only a few determinants seem relevant for explaining health app use among people with T2D, indicating a need for more future research on which people with T2D use health apps and why.

## Introduction

### Background

Type 2 diabetes (T2D) is a common chronic metabolic disease that increases the risk for severe health complications and premature death [1,2] and is the cause of high economic costs both in Germany and worldwide [3,4]. Thus, current numbers of adults with diabetes and those who are at high risk for developing diabetes are alarming, both worldwide and for the German population [5,6]. However, mirroring the rising trend of diabetes-related risk factors such as obesity, the number of people with T2D is expected to rise, not only in the older generations but also in young people [7,8].

Mobile apps addressing health issues (health apps) provide an effective opportunity to support individuals in the prevention of diabetes, ie, health behavior change in general [9-11] or lowering diabetes risk in people without diabetes and with prediabetes [12], and in managing diabetes and preventing its complications [13,14]. Smartphones, which enable the use of health apps, are widespread as 81% of the German population older than 14 years used a smartphone in 2017 [15], and smartphone use is still increasing [16]. Therefore, apps have the potential to save health care costs [17] and to reach many people, those with and without illness conditions. Despite the effects and potential benefits of general and diabetes-related health apps, less is known about who uses health apps, especially when focusing on people without known diabetes and people with T2D. The investigation of health app use and its association with a range of physiological, personal, and environmental factors representing a persons’ health and life background as conceptualized in the International Classification of Functioning, Disability, and Health model [18] among people with and without T2D may result in group-specific user characterizations. In turn, these may be valuable for needs-based and target group–specific health app development and health app promotion.

Although a few studies from a few countries exist that investigate the rates of health app use among the general population [19-22], research investigating the rates of smartphone and health app use among the general population having no diagnosed diabetes is scarce. This gap in the literature motivates the investigation of potential determinants relevant for prevention, ie, determinants that are only present in people without diabetes such as risk perception on developing diabetes. In the characterization of health app users among the general population, previous research consistently suggests that age is associated with health app use [19-23], whereas the investigation of sociodemographic factors such as gender or educational level revealed mixed results [19-23]. Besides electronic health (eHealth) literacy, health awareness, and health intentions [19,21,22], which seem to be correlated with health app use, previous studies focused on health-related behaviors [19-22] as they usually present the primary target of health apps. Evidence of the association with health app use was found for physical activity [19,21,22] but not for smoking [20,21], whereas it was unclear for BMI or obesity [20,21]. Thus, these factors, which are associated with the risk of developing T2D, shall be further explored alongside other factors that contribute to an actual diabetes risk, eg, as indicated by a diabetes risk test. However, an actual diabetes risk, can, but must not, reflect health beliefs. A previous study revealed that the perceived diabetes risk was low, even if the actual risk was high [24]. Thus, psychological and health factors should be explored in addition for the characterization of health app users. Furthermore, a healthy lifestyle or diabetes risk addressed by a health care professional has been found to be associated with adopting a healthy lifestyle [25,26]. This kind of taking care of ones’ own health, which Cho et al [27] refer to as health consciousness, was found to be associated with health app use. Thus, physician-communicated health information shall be explored in the context of health app use.

Research focusing on user rates and the identification of potential determinants of health app use in people with T2D seems to exist even less. Although there have been estimations of user rates among people with known T2D among the Australian adult population [28], such estimates seem to be generally lacking for the German population. Previous studies examined the potential determinants of health app use among people with chronic conditions, including diabetes [29,30]. However, these studies did not investigate people with T2D separately. Zhang et al [29] found lower health app use in patients with T2D compared with patients with type 1 diabetes (T1D), indicating the need for differentiated examinations of health app use for each diabetes type. Trawley et al investigated associations between app use for diabetes management and sociodemographic, clinical, and psychosocial factors among people with T1D and T2D. However, subsequent analyses of individuals with T2D were not conducted because of an insufficient sample size [28]. Previous research indicated that clinical indicators and disease-related factors may be associated with the usage of mobile health (mHealth) or eHealth technology. Usage seemed to be more relevant for people with a shorter diabetes duration [28,31]. Kuerbis et al [32] discussed disabilities and functional capacities as potential barriers of usage. Another study found that patients with T2D most often chose an app that could receive blood sugar data from a blood glucometer [29]. Control beliefs seem to be relevant for patients’ self-care behaviors [33] and thus might increase their likelihood to engage in health app use to improve diabetes management. To extend the literature on characteristics of health app users and nonusers among people with T2D, potential determinants of health app use, similar to those in people without diabetes but also more disease related, should be explored.

### Objectives

Identifying rates of smartphone ownership, health app use, and behavior types targeted by apps, as well as characterizing health app users and nonusers by using population-based data, might be helpful to promote health app use in specific health contexts such as diabetes prevention and management. To fill the gaps described earlier, this study aimed to provide user rates for adults without diabetes (ie, focusing on diabetes prevention) and for adults with T2D (ie, focusing on diabetes management) in Germany. For both samples, we particularly intended to explore associations between health app use and a range of potential determinants (partly group specific and partly overlapping between samples) that we summarized as sociodemographic factors, diabetes-related risk factors or indicators, psychological and health-related factors, and physician-provided information.

## Methods

### Study Design and Sample

The survey, *Disease knowledge and information needs—Diabetes mellitus (2017)*, was conducted in 2017 by the Robert Koch Institute (RKI), Berlin (Germany). This nationwide telephone survey focused on psychosocial and health care factors in adults without known diabetes and people with known diabetes. People were eligible to participate in the survey if they were German residents aged at least 18 years and had sufficient German language skills. For the survey, the aim was to include a sample of 2500 people without known diabetes and a sample of 1500 people with known diabetes to identify subgroups and to ensure stratified analyses with possibly low levels of error tolerance. The sampling procedure comprised a dual-frame approach. To ensure representativeness by considering all private households that were potentially reachable over the phone, a sample of landline and mobile telephone numbers was randomly generated. In a first step, a sample of the adult general population, including people with and without diabetes, was drawn using the Kish selection grid method. Assignment to one of the samples was based on the question “Have you ever been diagnosed with diabetes by a doctor?” (yes or no). Two respondents were excluded because of not answering the question with yes or no or because of missing information about the federal state of residence. To gain a larger sample of people with a physician-diagnosed diabetes, in a second step, a direct screening procedure was applied by asking for lifetime physician-diagnosed diabetes. More details are presented elsewhere [34]. Final samples comprised 2327 people without known diabetes and 1479 people with self-reported physician-diagnosed diabetes, respectively. Data were collected by an external market and social research institute between September and November 2017 applying computer-assisted telephone interviews. In this cross-sectional survey, all respondents were interviewed at a single point of measurement. Interviews were based on 2 different questionnaires, customized for people without and with diabetes. Questionnaires were developed by using preferably short and validated German-language instruments. English-language instruments used were translated into German language using the forward-backward translation [35]. Other questions were newly developed. Psychometric properties of multi-item measurements were investigated and will be the content of a separate publication that is currently under revision. Cognitive testing and a field pretest were conducted to assess the comprehensibility and length of the questionnaires. To ensure RKI quality standards of survey assessment, interviewers were trained, monitored, and supervised by the RKI [36]. Respondents’ willingness of cooperation with the survey was strengthened by applying interview options and rules (eg, making appointments with target persons; limited number of contact attempts).

This study focused on individuals without known diabetes (n=2327) and on individuals with self-reported T2D (n=1149). People who reported types other than T2D were excluded from this study (n=330). This study was reported by following the Strengthening the Reporting of Observational studies in Epidemiology guidelines for cross-sectional studies [37].

The survey was approved by the ethics committee of the Berlin Chamber of Physicians in August 2017 (Ärztekammer Berlin; number Eth-23/17) and the Federal Commissioner for Data Protection and Freedom of Information. All participants were informed about the voluntary nature of their participation and the survey objectives and provided oral informed consent to participate in the survey before the interview started.

### Survey Measures

#### Smartphone Ownership and Health App Use

Smartphone ownership (yes or no) was assessed identically in both samples by asking a single question. An overview of survey questions is given in Multimedia Appendix 1. Health app use, which was the dependent variable, was only assessed in people who stated they owned a smartphone. Participants of both samples were asked if they used a smartphone or app to improve a certain behavior in the last 12 months. Health behaviors listed were to (1) quit smoking, (2) be regularly physically active, (3) maintain a healthy diet, (4) reduce weight, (5) take medication regularly, (6) regulate blood pressure, and (7) regulate blood sugar (only in people with diabetes); this was adapted from the study by Ernsting et al [19]. People who stated that they use a smartphone or apps to improve at least one of the target behaviors were defined as health app users. Participants who answered *no* for all behaviors but used apps for behaviors not listed in the survey (derived from the question: “Is it correct that you do not use your smartphone or apps to improve behaviors?”) were defined as health app users as well.

#### Determinant Variables in People Without Known Diabetes

The sociodemographic determinants assessed were age (subsequently categorized into 18-44, 45-64, and ≥65 years), sex, and educational level. The latter was determined following the Comparative Analysis of Social Mobility in Industrial Nations classification system [38] and was categorized as low, middle, or high [6]. Diabetes risk factors considered in this study comprised BMI (in kg/m²) and several components of the German Diabetes Risk Score (GDRS [39,40]). BMI was calculated based on the participants’ self-reported body height and weight (kg/m²) and categorized into normal (BMI<25 kg/m²), overweight (BMI≥25 kg/m²), and obese (BMI≥30 kg/m²) based on the World Health Organization’s criteria [41]. The components of GDRS were hypertension diagnosis (yes or no), physical activity (more or less than 5 hours), smoking (current, former, or nonsmoker), and a family history of diabetes (ie, having at least one biological parent or sibling who was diagnosed with diabetes). Perceived health and presence of chronic diseases apart from diabetes were assessed with an item of the Minimum European Health Module [42] each. The perceived risk of getting diabetes over the next 5 years was assessed with an item adopted from a study by Kim at el [43]. Health advice obtained by a physician was assessed by asking those who had been at a medical practice in the last 12 months whether they had received advice on several health behaviors. Items were adapted from the German Health Interview and Examination Survey for Adults (DEGS1) [44]. Participants were defined as having obtained health advice if they stated that they had received advice on at least one health behavior. An increased diabetes risk communicated by a physician (yes or no) was assessed with a self-developed item.

#### Determinant Variables in People With Known Type 2 Diabetes

Age, sex, and educational level were assessed analogously for people without diabetes. Diabetes-related indicators included in this study were diabetes duration, BMI (kg/m^2^), diabetes-related complications, comorbidities, current diabetes treatment, and the method of blood sugar measurement. Diabetes duration was calculated based on self-reported age and the time of diagnosis. Diabetes-related complications and comorbidities were assessed with several items adopted from DEGS1 [44]. Participants were asked for their current diabetes treatment with an item adopted from the German Health Update survey [45]. The method of blood sugar measurement was assessed with a self-developed item. People were asked if they use a blood glucose meter and a glucose sensor in the subcutaneous fatty tissue, ie, continuous glucose monitoring systems and flash glucose monitoring systems. Perceived health was assessed using the same item as for participants without diabetes. Personal control over diabetes was assessed by using the Personal Control subscale of a German version of the Revised Illness Perception Questionnaire (IPQ-R) [46,47]. The scale score was calculated following instructions that were presented in the German IPQ-R downloaded from the IPQ-R website [47]. The scale had a possible range from 4 to 20, with a higher score reflecting more personal control. Health advice obtained by a physician was assessed similarly to participants without diabetes, ie, participants with diabetes were directly asked if they received advice on health behaviors by a member of their medical treatment team in the last 12 months.

### Statistical Analyses

All analyses were conducted separately for people without known diabetes and people with T2D. Logistic regression models, with health app use as the dependent variable, were performed only among those who stated that they owned a smartphone (sample without known diabetes n=1690; sample with T2D n=481). Participants who reported not owning a smartphone or not knowing were excluded from the analyses. For comparison, descriptive statistics and examinations comparing nonsmartphone users and smartphone users are presented in Multimedia Appendix 2. Separate models that included the single determinants only adjusted for age and sex were calculated for each sample (model 1). Then, a fully adjusted model, including all variables described above simultaneously, was calculated for each sample (model 2). Specific weighting factors were applied and calculated for people without and with diabetes, as previously described in more detail [34]. Logistic regression assumptions were tested, revealing no multicollinearity, no independence of residuals, and no linearity of continuous variables. The overall model fit was evaluated by the Hosmer-Lemeshow goodness-of-fit statistic [48] and the models’ discrimination ability, ie, the area under the receiver operating characteristic (ROC) curve [48] with a value of 0.7 indicating acceptable discrimination.

Missing data were treated by applying multiple imputation separately to each initial sample, ie, people without known diabetes (n=2327) and people with T2D (n=1148). In the sample with T2D, one case was excluded before the imputation because of missing information on smartphone ownership. Chained equation imputation was performed based on the fully conditional specification (FCS) method assuming the pattern of missing data to be arbitrary and by choosing 20 imputations [49]. Two imputation models were built, one for each sample, ie, people without and with T2D. A model included all corresponding sample variables used for the logistic regression, or items, which were required to build those variables and the corresponding sample weight. In the sample of people without diabetes (n=2327), 149 (6.4%) participants had missing information in at least one variable. All variables included in the imputation model had less than 5% of missing values. Precise information on missing values for all variables is presented in Multimedia Appendix 3. In the sample of people with T2D (n=1149), 206 (17.9%) participants had missing information in at least one variable. Missing data made up less than 5% per variable for all variables (Multimedia Appendix 3). Logistic regression was run separately in the 20 imputation data sets, resulting in combined parameter estimates. The mean Cronbach alpha across 20 imputed datasets was alpha=.79 for the personal control scale.

All analyses were performed by using the statistic software SPSS (IBM SPSS version 25.0). *P* values <.05 were considered to indicate statistical significance.

## Results

### Smartphone Ownership and Health App Use

**Figure 1:**
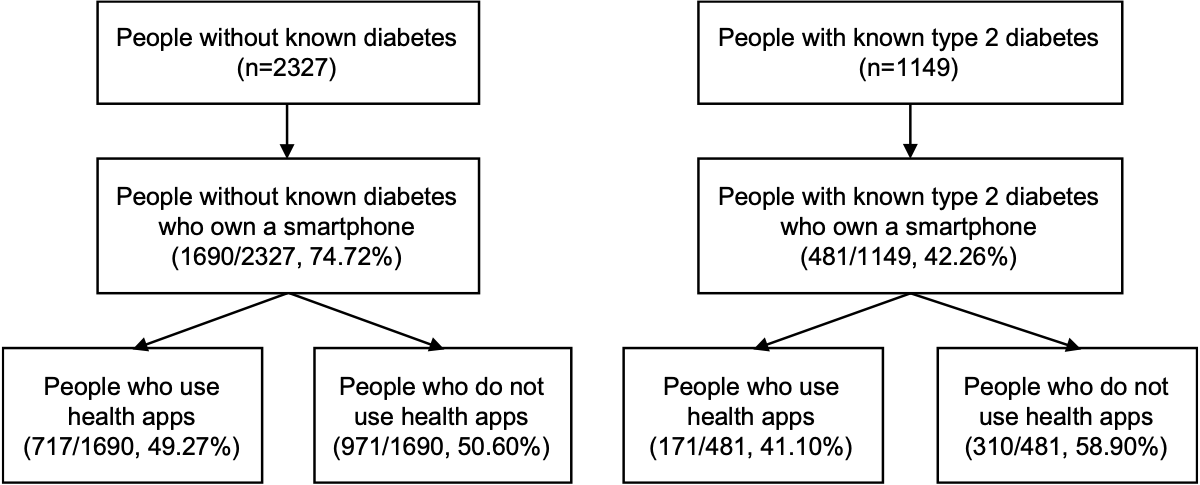
A Flowchart of the hierarchical sample structure for people without known diabetes and people with known type 2 diabetes. Sample sizes (n) are given as unweighted data. Percentages are given as weighted.

Within the initial sample of people without known diabetes (n=2327), the majority reported to own a smartphone (1690/2327, 74.72%; Figure 1). Among smartphone owners, ie, the analysis sample in this study, the mean age, after weighting, was 43.7 (SD 15.7) years, ranging from 18 to 91 years. The proportion of women and men, after weighting, was comparable (887/1690, 50.78% vs 860/1690, 49.22%, respectively; Table 1). Among those who owned a smartphone (n=1690), about half of the participants (717/1690, 49.27%, which was 36.81% of the initial sample n=2327) reported using apps to improve health behaviors (Figure 1). Sample characteristics of the initial sample, smartphone owners, app users, and nonapp users without known diabetes are presented in Table 1.

**Table 1:** Sample characteristics of the initial sample and of the sample of smartphone owners, app users, and nonapp users among individuals without known diabetes. (Data are given as weighted percentage or mean [SD]. Missing data were less than 5% for all variables).

Variables			Initial sample (n=2327)	Smartphone owners^a^ (1690/2327; 74.72%^b^)	App users^a^ (717/1690; 49.27%)	Nonapp users^a^ (971/1690; 50.60%)
**Sociodemographic factors**						
	Age (years), mean (SD)		49.62 (18.55)	43.69 (15.73)	40.78 (15.06)	46.50 (15.88)
	**Age (years), %**					
		≥65	22.57	9.79	6.08	13.43
		45-64	36.68	37.76	32.71	42.53
		18-44	40.76	52.45	61.22	44.05
	Gender, female (%)		51.68	50.78	50.49	50.94
	**Educational level (%)**					
		Low	30.70	23.61	22.58	24.68
		Middle	42.16	45.48	49.49	41.69
		High	26.93	30.63	27.51	33.49
**Diabetes-related risk factors**						
	BMI (kg/m²), mean (SD)		25.61 (4.57)	25.38 (4.50)	25.59 (4.47)	25.19 (4.52)
	**BMI (kg/m²; %)**					
		BMI<25	50.55	53.31	49.71	56.70
		25≤BMI<30	31.52	29.59	32.00	27.33
		BMI≥30	15.85	15.07	16.24	13.97
	Hypertension diagnosis (%)		32.60	25.82	25.75	25.96
	Physical activity ≥5 hours/week (%)		72.49	73.46	74.28	72.59
	**Smoking (%)**					
		Not smoking	47.91	45.13	40.25	49.74
		Former smoking	24.69	24.29	26.05	22.65
		Currently smoking	27.40	30.58	33.70	27.62
	Family history of diabetes (%)^c^		22.29	21.34	21.88	20.88
**Psychological and health-related factors**						
	**Perceived health (%)**					
		Very good	24.96	28.30	33.11	23.69
		Good	48.77	50.25	47.22	53.07
		Moderate/poor/very poor	26.23	21.44	19.66	23.23
	Chronic diseases (%)^d^		43.86	40.65	41.30	39.86
	**Perceived risk of getting diabetes (%)**					
		Almost no risk	42.00	41.37	44.92	37.86
		Slight risk	39.41	40.91	39.18	42.69
		Moderate risk	11.52	11.88	12.72	11.00
		High risk	2.27	2.52	1.26	3.76
**Physician-provided information (%)**						
	Health advice obtained by physician		46.75	48.30	52.20	44.46
	Diabetes risk communicated by physician		6.09	5.89	5.12	6.56

^a^Sample sizes (n) are given as unweighted.

^b^Percentages (%) are given as weighted.

^c^At least one parent or sibling was diagnosed with diabetes.

^d^Any chronic disease besides diabetes.

**Figure 2:**
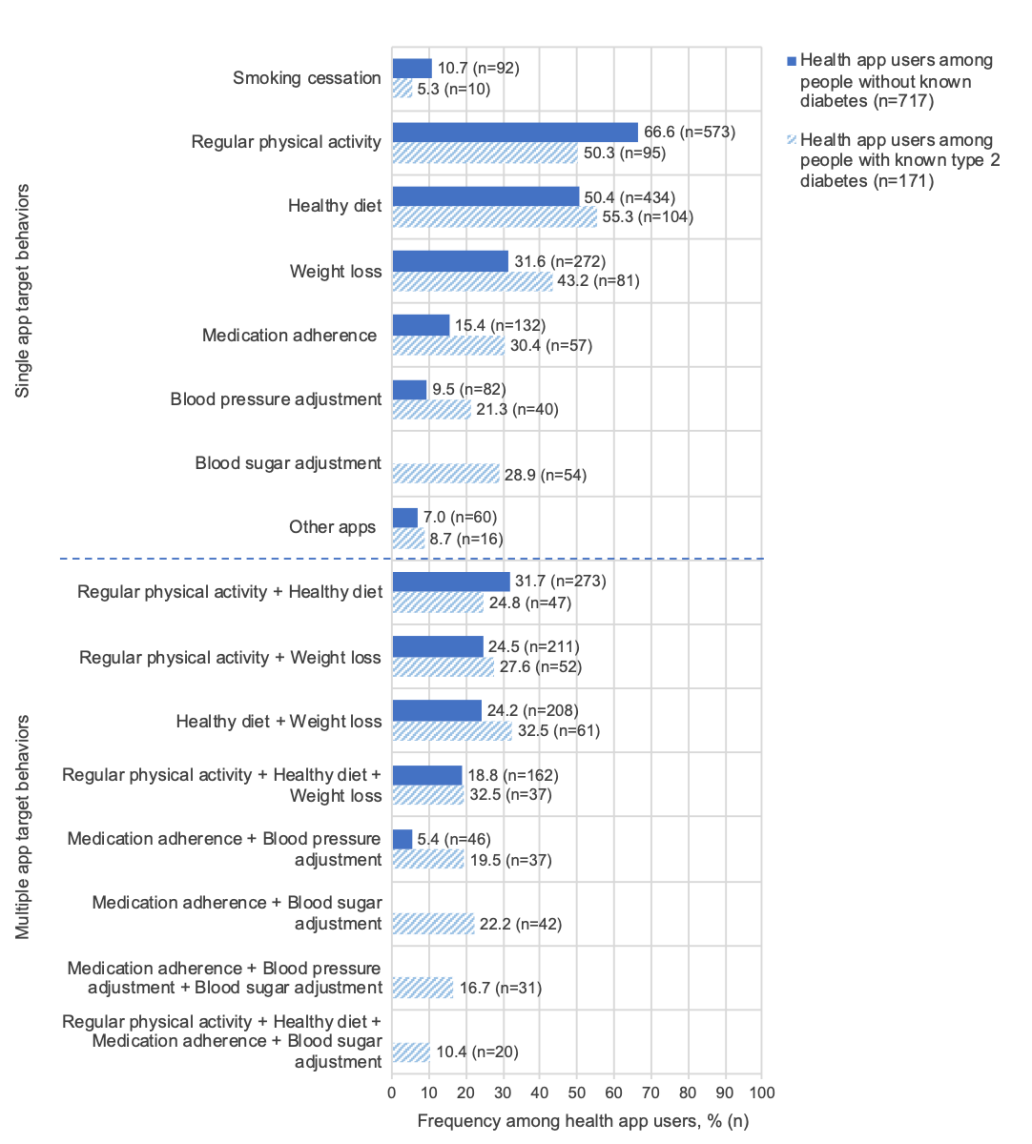
App use for single and multiple target behaviors among people without known diabetes and people with known type 2 diabetes. Frequencies are given as weighted percentage and n. App use targeting blood sugar adjustment was assessed only in people with type 2 diabetes and thus is not presented for people without known diabetes in the single and multiple condition.

Within the initial sample of people with known T2D (n=1149), less than half of the participants reported owning a smartphone (481/1149, 42.26%; Figure 1). In smartphone owners, the mean age, after weighting, was 61.6 (SD 11.7) years, ranging from 18 to 95 years. In this sample, after weighting, 43.6% (200/481) were female (Table 2). Among those who owned a smartphone (n=481), 171 participants (41.1%, ie, 17.4% of the initial sample, n=1149) used apps to improve health behaviors (Figure 2). Sample characteristics of the initial sample, smartphone owners, app users and nonapp users with known T2D are presented in Table 2.

### Health Behaviors Targeted by Apps

When considering single health behaviors targeted by apps among health app users without known diabetes (n=717), the most frequent target behaviors, after weighting, were improving physical activity (573/717, 66.6%), healthy diet (434/717, 50.4%), and weight loss (272/717, 31.6%; Figure 2). Apps were used least frequently for blood pressure adjustment (82/717, 9.5%), smoking cessation (92/717, 10.7%), and medication adherence (132/717, 15.4%). When focusing on combinations of multiple health behaviors targeted by apps (Figure 2), regular physical activity and healthy diet were most often reported to be simultaneously addressed by apps (273/717, 31.7%). Among health app users with T2D (n=171), single health behaviors targeted by apps most often were a healthy diet (104/171, 55.3 %), regular physical activity (95/171, 50.3%), and weight loss (81/171, 43.2%; Figure 2). App use was reported less often for medication adherence (57/171, 30.4%), blood sugar adjustment (54/171, 28.9%), blood pressure adjustment (40/171, 21.3%), and smoking cessation (10/171, 5.3%). The most frequent combination of multiple behaviors targeted by apps comprised a healthy diet and weight loss (61/171, 32.5%; Figure 2). Very few of the participants (20/171, 10.4%) reported using apps for a combination of regular physical activity, a healthy diet, medication adherence, and blood sugar adjustment.

**Table 2:** Sample characteristics of the initial sample and of the sample of smartphone owners, app users, and nonapp users among individuals with known type 2 diabetes. (Data are given as weighted percentage or mean [SD]. Missing data were less than 5% for all variables).

Variables				Initial sample (n=1149)		Smartphone owners^a^ (481/1149; 42.26%^b^)		App users^a^ (171/481; 41.10%)		Nonapp users^a^ (310/481; 58.90%)	
**Sociodemographic factors**											
	**Age (years), mean (SD)**			67.37 (12.06)		61.64 (11.73)		58.80 (13.95)		63.61 (9.43)	
	**Age (years), %**										
			≥65	62.35		41.89		31.07		49.55	
			18-64	37.65		58.11		68.93		50.45	
	Gender, female (%)			50.95		43.58		51.01		38.40	
	**Educational level (%)**										
			Low	49.15		37.19		33.94		39.45	
			Middle	38.35		46.28		52.22		42.13	
			High	12.43		16.54		13.84		18.42	
**Diabetes-related indicators**											
	Diabetes duration (years), mean (SD)			13.78 (9.75)		11.79 (8.61)		11.12 (8.41)		12.25 (8.74)	
	BMI (kg/m²), mean (SD)			30.53 (5.78)		30.81 (6.11)		30.59 (6.39)		30.97 (5.92)	
	**BMI (kg/m²; %)**										
			BMI<25	15.25		15.51		19.12		12.99	
			25≤BMI<30	35.61		33.25		30.18		35.40	
			BMI≥30	46.78		50.15		49.70		50.47	
	Diabetes-related complications^c^, at least one (%)			34.33		32.23		30.77		33.26	
	Comorbidities^d^, at least one (%)			31.01		24.42		20.96		26.84	
	Treatment with insulin (%)			45.54		41.05		46.08		37.54	
	Treatment with tablets (%)			69.81		75.02		76.38		74.08	
	Treatment with healthy diet or physical activity (%)			73.18		78.94		83.29		75.91	
	**Method of blood sugar measurement^e^ (%)**										
		Glucose meter with blood sampling			62.12		61.35		61.78		61.05
		Subcutaneous fatty tissue in addition to or instead of a glucose meter			4.96		5.10		8.34		2.84
		No use of measurements or no blood sugar measuring in the last 7 days			31.75		32.99		28.94		35.82
**Psychological and health-related factors**											
	**Perceived health (%)**										
		Very good			6.51		8.82		8.92		8.74
		Good			40.78		43.35		43.52		43.23
		Moderate/poor/very poor			52.58		47.78		47.56		47.93
	Personal control over diabetes, mean (SD)^f^			16.02 (2.79)		16.86 (2.67)		16.87 (2.95)		16.85 (2.47)	
**Physician-provided information**											
	Health advice obtained by physician (%)			81.91		83.60		86.53		81.56	

^a^Sample sizes (n) are given as unweighted.

^b^Percentages (%) are given as weighted.

^c^Including kidney disease, eye disease, nervous disease, diabetic foot lesions, and amputations.

^d^Including heart attack, stroke, and coronary heart disease.

^e^Multiple answers were eligible.

^f^Possible score range: 4-20.

### Factors Associated With Health App Use

Logistic regression analyses among people without known diabetes who reported to own a smartphone (n=1690) revealed almost consistent patterns of results when comparing the age- and sex-adjusted models (model 1) with the fully adjusted model (model 2), except for educational level and perceived risk of getting diabetes (Table 3). For model 2, the Hosmer and Lemeshow test revealed a nonsignificant result (χ²_8_=13.8; *P*=.16), indicating that the model fits the data. The area under the ROC curve (0.68) indicated acceptable discrimination.

**Table 3:** Associations with app use among people without known diabetes owning a smartphone (n=1690). Analyses were based on imputed and weighted data. Model 1: adjusted for age and gender. Model 2: fully adjusted for all determinants. Model statistics for model 2 (values were averaged based on 20 imputed datasets): Hosmer and Lemeshow test: χ²8=13.8; *P*=.16; area under the receiver operating characteristic curve=0.68.

Factors			Model 1					Model 2			
			Odds ratio (95% CI)			*P* value		Odds ratio (95% CI)		*P* value	
**Sociodemographic factors**											
	**Age (years)^a^**										
		≥65		—^b^			—		—		—
		45-64		1.74 (1.21-2.50)			.003		1.76 (1.19-2.59)		.004
		18-44		3.07 (2.16-4.36)			<.001		3.89 (2.62-5.78)		<.001
	Gender (reference: male)^a^			0.96 (0.79-1.16)			.63		1.04 (0.85-1.29)		.69
	**Educational level**										
		Low		—			—		—		—
		Middle		1.07 (0.83-1.38)			.61		1.23 (0.94-1.61)		.13
		High		0.74 (0.56-0.97)			.03		0.86 (0.64-1.15)		.30
**Diabetes-related risk factors**											
	**BMI (kg/m^2^)**										
		BMI<25		—			—		—		—
		25≤BMI<30		1.56 (1.24-1.96)			<.001		1.58 (1.24-2.02)		<.001
		BMI≥30		1.84 (1.35-2.51)			<.001		2.07 (1.45-2.96)		<.001
	Hypertension diagnosis (reference: no)			1.45 (1.14-1.85)			.003		1.31 (1.01-1.70)		.045
	Physical activity (≥5 hours per week; reference: <5 hours)			1.10 (0.87-0.137)			.43		1.00 (0.79-1.28)		.97
	**Smoking**										
		Not smoking		—			—		—		—
		Formerly smoking		1.66 (1.30-2.12)			<.001		1.51 (1.17-1.96)		.002
		Currently smoking		1.54 (1.22-1.94)			<.001		1.58 (1.24-2.01)		<.001
	Family history of diabetes (reference: no)			1.17 (0.93-1.49)			.19		1.29 (1.00-1.66)		.06
**Psychological and health-related factors**											
	**Perceived health**										
		Moderate/poor/very poor		—	—		—		—		—
		Good		0.91	0.70-1.18		.47		1.22 (0.90-1.64)		.20
		Very good		1.28	0.96-1.71		.10		2.21 (1.55-3.16)		<.001
	Chronic diseases (reference: no)			1.27	1.04-1.56		.02		1.48 (1.16-1.88)		.002
	**Perceived risk of getting diabetes**										
		Almost no risk		—	—		—		—		—
		Slight risk		0.85	0.68-1.06		.14		0.78 (0.62-0.99)		.04
		Moderate risk		1.07	0.78-1.50		.67		0.92 (0.64-1.33)		.67
		High risk		0.29	0.14-0.60		.001		0.23 (0.10-0.51)		<.001
**Physician-provided information**											
	Health advice obtained by physician (reference: no)			1.49	1.23-1.81		<.001		1.48 (1.20-1.83)		<.001
	Diabetes risk communicated by physician (reference: no)			0.82	0.54-1.23		.34		0.69 (0.43-1.11)		.13

^a^The separate model was not adjusted for any other variable.

^b^Reference group.

Among people without known diabetes, results of model 2 (fully adjusted) revealed that younger and middle-aged participants (18-44 and 45-64 years) were more likely to use health apps compared with older participants (≥65 years; OR 3.89; *P*<.001; OR 1.76; *P*=.004). Sex and educational level were not significantly associated with health app use (Table 3).

Overweight or obese participants were more likely to be health app users compared with participants with a normal BMI (OR 1.58; *P*<.001; OR 2.07; *P*<.001). Participants who had been diagnosed with hypertension were more likely to use apps compared with participants who never had a hypertension diagnosis (OR 1.31; *P*=.045). Current and former smokers were more likely to use health apps compared with nonsmokers (OR 1.58; *P*<.001; 1.51; *P*=.002). Regular physical activity and having a family history of diabetes were the only risk factors that were not found to be significantly associated with health app use.

All psychological and health-related factors were found to be associated with health app use in model 2. Participants who perceived their health as very good were more likely to use health apps compared with participants with poorer self-perceived health (moderate to very poor; 2.21; *P*<.001). However, this difference could not be found for those who perceived their health as good. Participants with a chronic disease were more likely to use health apps than those without chronic disease (OR 1.48; *P*=.002). Participants who perceived themselves at a slight or high risk of getting diabetes in the next 5 years were less likely to use health apps compared with those who perceived themselves at almost no risk (OR 0.78; *P*=.04; OR 0.23; *P*<.001). However, this could not be observed for participants who perceived their risk as moderate.

Considering physician-provided information factors, participants who obtained health advice from a physician were more likely to use health apps compared with those who received no advice on any health behavior (OR 1.48; *P*<.001). A present diabetes risk communicated by a physician was not associated with health app use.

Subsequent logistic regression analyses exploring potential associations of smartphone ownership among people without diabetes revealed similar results as found for health app use (Multimedia Appendix 2). Remarkably, educational level and physical activity were found to be significantly associated with smartphone ownership, but BMI, chronic diseases, and perceived risk of getting diabetes were not.

Among people with T2D who reported owning a smartphone (n=481), the fully adjusted logistic regression analysis revealed similar results compared with results based on the age- and sex-adjusted models for each determinant (Table 4). Regarding model 2, the Hosmer and Lemeshow test revealed a nonsignificant result (χ²_8_=9.4; *P*=.33), indicating that the model fits the data. The area under the ROC curve (0.69) indicated acceptable discrimination.

In model 2 (fully adjusted), participants between 18 and 64 years of age were more likely to use apps compared with participants who were 65 years or older (OR 1.84; *P*=.007). Compared with men, women were more likely to use health apps (OR 1.61; *P*=.02). Of the remaining potential determinants, only the method of blood sugar measurement was associated with health app use. Participants who used both a glucose meter with blood sampling and a blood glucose sensor in the subcutaneous fatty tissue were more likely to use health apps compared with participants who only used a glucose meter with blood sampling (OR 2.74; *P*=.04). 

Results of subsequent logistic regression analyses exploring potential associations of smartphone ownership among people with T2D were similar compared with those found for health app use (Multimedia Appendix 2). However, educational level, diabetes duration, perceived health, and personal control over diabetes were found to be significantly associated with smartphone ownership, whereas no association was found for the method of blood sugar measurement.

**Table 4:** Associations with app use among people with type 2 diabetes owning a smartphone (n=481). Analyses were based on imputed and weighted data. Model 1: adjusted for age and gender. Model 2: fully adjusted for all determinants. Model statistics for model 2 (values were averaged based on 20 imputed datasets). Hosmer and Lemeshow test: χ²8=9.4; *P*=.33; area under the receiver operating characteristic curve=0.69.

Factors			Model 1				Model 2			
			Odds ratio (95% CI)		*P* value		Odds ratio (95% CI)		*P* value	
**Sociodemographic factors**										
	**Age (years)^a,b^**									
		≥a		—^c^		—		—		—
		18-64		2.16 (1.47-3.19)		<.001		1.84 (1.19-2.86)		.007
	Gender (reference: male)^b^			1.68 (1.15-2.44)		.007		1.61 (1.07-2.43)		.02
	**Educational level**									
		Low		—		—		—		—
		Middle		1.36 (0.89-2.06)		.16		1.47 (0.95-2.21)		.09
		High		0.97 (0.72-1.30)		.98		1.10 (0.63-2.01)		.75
**Diabetes-related indicators**										
	Diabetes duration (years)			1.00 (0.97-1.02)		.72		0.99 (0.96-1.01)		.26
	**BMI (kg/m^2^)**									
		BMI<25		—		—		—		—
		25≤BMI<30		0.62 (0.34-1.11)		.11		0.63 (0.34-1.17)		.14
		BMI≥30		0.60 (0.34-1.05)		.07		0.62 (0.34-1.12)		.11
	**Diabetes-related complications^d^**									
		At least one complication (reference: no complication)		0.98 (0.65-1.48)		.92		0.97 (0.61-1.54)		.89

	**Comorbidities^e^**									
		At least one comorbidity (reference: no comorbidity)		0.89 (0.56-1.42)		.63		0.87 (0.53-1.43)		.59
	Treatment with insulin (reference: no)			1.45 (0.98-2.14)		.06		1.53 (0.92-2.55)		.11
	Treatment with tablets (reference: no)			1.05 (0.68-1.63)		.82		1.10 (0.67-1.79)		.71
	Treatment with healthy diet or physical activity			1.56 (1.24-1.96)		.07		1.58 (0.94-2.68)		.09
	**Method of blood sugar measurement**									
		Glucose meter with blood sampling		—		—		—		—
		Blood glucose sensor in subcutaneous fatty tissue in addition to or instead of a glucose meter		2.50 (1.00-6.24)		.05		2.74 (1.06-7.09)		.04
		No use of measurements or no blood sugar measuring in the last 7 days		0.76 (0.50-1.16)		.20		0.85 (0.52-1.38)		.50
**Psychological and health-related factors**										
	**Perceived health**									
		Moderate/poor/very poor		—		—		—		—
		Good		1.06 (0.71-1.58)		.78		1.01 (0.65-1.58)		.97
		Very good		0.90 (0.45-1.81)		.78		1.01 (0.49-2.10)		.98
	Personal control over diabetes			0.97 (0.90-1.04)		.40		0.98 (0.91-1.06)		.60
**Physician-provided information**										
	Health advice obtained by physician (reference: no)			1.39 (0.81-2.39)		.23		1.28 (0.72-2.28)		.40

^a^The separate model was not adjusted for any other variable.

^b^Age categories *18-44* and *45-64* years were merged because of insufficient case numbers in the age category *18-44* years across other variables.

^c^Reference group.

^d^Complications asked in this survey were kidney disease, eye disease, nervous disease, diabetic foot lesions, and amputations.

^e^Comorbidities asked in this survey were heart attack, stroke, and coronary heart disease.

## Discussion

### Principal Findings

On the basis of data from a nationwide telephone survey targeting the German adult population, about 75% of people without known diabetes, ie, those who may be targets for diabetes prevention interventions, owned a smartphone. Among those, every second person used health apps. However, in people with known T2D, ie, who could be considered potential recipients of diabetes management interventions, about 40% of the diabetes sample owned a smartphone. Less than 3 out of 10 smartphone owners used health apps.

In people without known diabetes, results suggested a correlation of health app use with several determinants including age; diabetes risk factors; and psychological and health factors such as perceived health, chronic diseases, perceived risk, and medical health advice. However, in people with T2D, only a few correlates of health app use were identified including age, sex, and method of blood sugar measurement.

### Strengths and Limitations

An essential strength of this study was the underlying nationwide survey of the German adult population covering both people without and with diabetes. Hence, the results of this study provided rates of smartphone ownership and updated rates of health app use as well as behaviors targeted by apps for the German population aged 18 years and above. However, only people with sufficient knowledge of the German language were eligible to participate in the survey. As a result, the survey data were not representative for people who do not speak German fluently, such as people with a recent history of migration. Moreover, as the survey mode comprised telephone interviews, a selected responsiveness to telephone calls and attendance in the survey cannot be ruled out, although sample weights were used to optimize representativeness. This study aimed to extend the literature on the characterization of health app users, which was previously addressed by only a few studies from a few countries. A wide range of determinants related to health app use were identified, contributing to a broader characterization of health app users and nonapp users among the general population without diabetes. However, the cross-sectional design did not allow for the investigation of causal relations, which should be investigated in subsequent research. Unfortunately, we were not able to find a similar range of determinants related to health app use among people with T2D. For instance, other factors that seem to influence the usage intention of telemedicine for diabetes management, eg, social influence or perceived ease of use [50], might play a more prominent role in predicting health app use. Nevertheless, we provided initial hints on population-based associations of actual health app use and gender, as well as the method of blood sugar measurement, in those with T2D. A limitation of the study is that findings may have been subject to biases because of self-reported data. Moreover, health app use as defined in this study, ie, using a smartphone or apps to improve health behaviors, may have differed from other definitions that, eg, referred to apps that were downloaded or categorized as health apps by common app stores. However, as this study focused on those who have engaged in health behavior improvements by using smartphone features or apps, our self-reported data still represent valuable insights.

### Comparison With Prior Work

#### Smartphone Ownership, Health App Use, and Target Behaviors

The rate of smartphone owners among people without diabetes found in this study (1690/2327, 74.72%) was comparable with rates found for the German adult population (72%) in the Global Attitudes Survey conducted in 2017 by the Pew Research Center [51]. The proportion of health app users among people without diabetes owning smartphones in our study (717/1690, 49.27%) was found to be about twice as high compared with previous surveys conducted in Germany in 2015 (age >35 years) [19], in the United States in 2013, and in China in 2016 (age >18 years) [21,22], but lower than the one found in an US survey conducted in 2015 (58%; sample age range 18-81 years) [20]. In our study, the proportion of health app users among people with T2D owning a smartphone was 41.1% (171/481), whereas it was 8% in a 2015 Australian study examining people who reported to own suitable devices to access apps or who had internet access and who used apps to manage their diabetes (sample age range 30-75 years) [28].

#### Health Behaviors Targeted by Apps

In line with the results of this study in people without known diabetes, prior findings of US studies revealed physical activity, diet or food tracking, and weight to be the most frequent behaviors targeted by apps [20,52].

Among people with T2D, the 3 most frequently reported health behaviors targeted by health apps were the same as in people without known diabetes. App use for blood sugar adjustment was reported by only 28.9% (54/171) of people with T2D in this study. In contrast, for Australia, Trawley et al [28] showed that 69% (18/26) and 57% (21/37) of people with T2D using insulin and not using insulin, respectively, used apps for recording their blood glucose levels. Although previous studies investigated whether diabetes management apps incorporated features according to the 7 self-care behaviors [53], this study revealed complementary results from a user perspective. Results showed that only 10.4% (20/171) of people with T2D used apps to simultaneously target regular physical activity, healthy diet, medication adherence, and blood sugar adjustment, all of which are part of the 7 self-care behaviors relevant for diabetes self-management [54].

#### Factors Associated With Health App Use

In people without known diabetes, the association of health app use and age found in this study was consistent with the results of various previous studies [19,20,22,55] contributing to the evidence of a higher health app relevance among younger people. Although previous studies from the United States [20,22,55] and China [21] have shown that health app use was associated with higher education, this study did not. This, however, is in accordance with other recent studies from Germany [19,23]. Concerning diabetes-related risk factors, results suggested health app use to be associated with BMI, smoking status, and hypertension diagnosis, but not with a family history of diabetes. Krebs et al also found app use to be more likely among people whose BMI are in the obese range [20]. However, previous studies did not find health app use or download to be associated with smoking status [19,21,55], a history of high blood pressure or cholesterol, or having a family member with diabetes [55]. Although physical activity was not found to be associated with health app use in this study, previous studies identified this association [19,21,22]. Among psychological and health factors in people without known diabetes, a better perceived health and having a chronic disease were associated with a more frequent health app use, similar to findings of prior research [19,21,22,30]. Regarding physician-provided information, results of this study revealed that people without diabetes who obtained health advice from a physician were more likely to use health apps compared with those who did not. Bender et al [55] found no association between health app download and discussing diabetes with a provider.

Among those with known T2D, we found health app use to be more likely in younger people compared with older people and in women compared with men. A recent survey from China found age differences in app use among people with any type of diabetes [29]. A larger proportion of female app users among those with T2D was found in a US survey from 2012 among the general population [52]. Other studies from Germany, Australia, and China did not find gender differences in app use in samples of older adults without diabetes [23], in people with T1D [28], and in people with any type of diabetes [29].

### Interpretation of the Findings and Practical Implications

The comparatively high rates of smartphone ownership and health app use in people without diabetes based on a nationwide survey point toward the increasing relevance of health apps. However, these rates were low among people with T2D. The low rate of health app use can partly be explained by the high mean age of people with T2D in our study, which in turn is related to low mobile phone ownership that may drive this effect. Indeed, advanced age has been found to be associated with a lower likelihood for owning a smartphone [19,22]. Moreover, recent studies on diabetes patients found that most patients did not use diabetes apps but were in need of a good app or interested in trying an app [56,57]. However, not knowing that diabetes apps exist seemed to be the main reason for not using an app [56,57]. Other studies indicated that the usability of apps for patients with chronic diseases, including diabetes, was not satisfying and caused frustration in patients [58,59]. However, diabetes seems to be an attractive field for digital health developments with high market potential in the future [60]. To provide all possible benefits to people with T2D, efforts to increase patients’ awareness of health apps are needed. In addition, developments of apps or smartphones should aim to increase adoption of patients by improving safety, effectiveness, interoperability with other tools, and compliance with clinical guidelines [58].

People without known diabetes, who may be considered as a potential target group for future diabetes prevention, and people with known T2D, who are the focus of complication prevention and diabetes management interventions, seem to have different health app use patterns. This may indicate specific preferences or needs for different health apps. Nevertheless, physical activity, a healthy diet, and weight loss were commonly sought app themes for both samples. In people without diabetes, health app use is likely not driven by the aim to prevent diabetes, but by motives related to general health improvement, illness avoidance, fitness, or appearance [61]. This may explain our findings of health app use being positively associated with behavioral diabetes-related risk factors (eg, smoking, a higher BMI, or hypertension) but being negatively associated with perceived risk of developing diabetes. Focusing on people with T2D who are already managing their diabetes, only 10.4% (20/171) of them used apps to simultaneously target 4 of the 7 self-care behaviors [62]. The explanation for this low rate may be twofold. On the one hand, the user either may just not perform multiple self-care behaviors or may have no need for app support to address multiple self-care behaviors. On the other hand, there might be a lack of convenient diabetes apps that target all self-care behaviors at once, as most diabetes management apps target only 2 to 3 self-care behaviors [53]. Developing apps that incorporate all self-care behavior domains may simplify and, thereby, encourage health app use for diabetes self-management.

Although health app use seemed not to be restricted to the higher educated in Germany, older age seemed to be an adverse factor when it comes to health app use, regardless of T2D diagnosis. Hence, future health interventions and health app developers should promote and support health app use among older people by considering age-related aspects such as age-specific design features, intuitive proceedings, easily understood training manuals, or presenting the clear purpose of the technology as health improvement [32]. In contrast to age and educational level, gender seemed to be a relevant correlate of health app use in people with T2D but not in people without diabetes. Guo et al [63] found that threat appraisal factors had a stronger effect on the attitude toward adoption of mHealth among women. As a health threat may be more present in people with a disease like diabetes, this gender affect may have appeared in people with T2D. However, as gender differences for health app use were not found in other samples with chronic diseases, including diabetes [28,29,64], more research will be required to clarify this point.

Our findings that people with an increased risk for diabetes, assessed by several diabetes risk factors, were more likely to use health apps seems promising. The increased health app use in those with an elevated risk might reflect an increased health consciousness [27] and the motivation to improve health behaviors. Moreover, the results suggest that those who have the greatest need for health behavior change and who might benefit the most are using health apps already. A next step may involve user’s guidance for choosing health apps, where health apps that have proven to be effective in achieving health improvements should be favored. Surprisingly, physical activity was not found to be associated with health app use. Different assessments of physical activity may explain the different results. We assessed physical activity according to the GDRS [39], ie, being physical active for less than 5 hours or 5 or more hours per week, whereas other studies used a cutoff of 2.5 hours or lower [19,22]. This indicates that the lower cutoff may be more applicable to explain health app use.

Our findings further indicate that it may be necessary to consider psychological factors when encouraging people to use health apps. Among people without known diabetes, those with an elevated perceived risk of getting diabetes were less likely to use health apps compared with those who perceived themselves at almost no risk. At first glance, this seems contradictive, as most social cognitive theories assume that a perceived health risk increases the likelihood of health-related preventive actions [65]. However, health app use presents only one possibility of support to improve health behaviors. Participants in this study were not asked for alternative strategies to improve health. Thus, those with high perceived risk might engage in health behavior change by using other strategies than apps. Moreover, the higher likelihood of health app use in participants with almost no perceived risk might be explained by the risk reappraisal hypothesis that suggests that the adoption of a preventive behavior reduces the perceived personal risk [66]. Thus, participants using health apps and maybe even acting preventive may, in turn, perceive themselves at low risk. Finally, high perceived risk but low perceived self-efficacy may lead to avoidance coping, which may also be linked with low app use rates, as shown in a study on health media use [67]. However, these hypothesizes cannot be tested in cross-sectional designs. Thus, future research including longitudinal designs might help to understand the association of health app use and risk perception.

The results of this study show that encouragement from health care providers to use health apps seems to be promising among people without known diabetes. Participants who received advice from a physician may have been more likely to use health apps because physicians may have recommended apps during health counseling. About 37% of physicians and about 40% of diabetologists were found to recommend health or diabetes apps [29,68]. Physicians who give advice on health behaviors may play an important role with the opportunity to encourage health app use among patients, but they should be supported by tools and guidelines to recommend appropriate apps [69]. However, as physician-communicated diabetes risk was not associated with health app use, the specific underlying mechanisms of the patient-provider context that promote health app use in patients need to be better understood.

### Conclusions

The data of a German population–based survey reflect that among people without known diabetes about every third person used health apps, whereas health apps were used by almost every seventh person in people with known T2D. A better understanding of the reasons that may explain this discrepancy may be addressed by future studies. Efforts to increase health app use in both people without and with T2D should keep in mind potential barriers to smartphone and health app use among older generations. Among people with T2D, in addition to age, we found that only a few determinants seem to be associated with health app use. Moreover, in people without known diabetes, diabetes-related risk factors and psychological and health factors should be considered for future target group–specific health app development. Importantly, our findings point out that health app use seems to be less likely when the perceived diabetes risk is high, but physicians’ health advice may play an important role in increasing health app use in patients.

## Appendix

Multimedia Appendix 1 Survey questions for people without known and with diabetes.

Multimedia Appendix 2 Descriptive statistics and examinations comparing nonsmartphone users and smartphone users.

Multimedia Appendix 3 Precise information on missing values for all variables included in the study.

## Abbreviations

DEGS1: German Health Interview and Examination Survey for Adults
eHealth: electronic health
FCS: fully conditional specification
GDRS: German Diabetes Risk Score
IPQ-R: Revised Illness Perception Questionnaire
mHealth: mobile health
RKI: Robert Koch Institute
ROC: receiver operating characteristic
T1D: type 1 diabetes
T2D: type 2 diabetes
