# Supplementary material for: Health App Use and Its Correlates Among Individuals With and Without Type 2 Diabetes: Nationwide Population-Based Survey
Source: JMIR Diabetes. 2020 May 20;5(2):e14396. doi: 10.2196/14396 (PMC7270854; doi:10.2196/14396)
Supplement: Multimedia Appendix 1 [file diabetes_v5i2e14396_app1.pdf]

1. Survey questions for both, respondents without known and with diabetes

Known diabetes

Have you ever been diagnosed with diabetes by a doctor?

Possible answers: yes, no

Smartphone ownership and health app use

1) Do you own a smartphone, that is, an internet-compatible cell phone?

Possible answers: yes, no

Respondents who own a smartphone were asked about app use.

2) Think about the last 12 months. Did you use your smartphone or apps, that is, applications or features of your smartphone to improve one of the following behaviors?

- Quit smoking
- Be regularly physically active
- Maintain a healthy diet
- Reduce weight
- Take medication regularly
- Regulate blood pressure
- Regulate blood sugar (only presented to respondents with known diabetes)

Possible answers: yes, no (multiple answers were possible)

Respondents who answered "no" regarding all the above mentioned health behaviors

3) Is it correct that you do not use your smartphone or apps to improve behaviors?

Possible answers: yes, no

(adopted from Ernsting et al. [1])

Perceived health

How is your health in general?

Possible answers: very good, good, fair, bad, very bad

(item of the Minimum European Health Module; MEHM [2])

Diabetes risk factors and diabetes risk

Body Mass Index (BMI)

1) How tall are you? (in cm)
2) How much do you weigh? (in kg)

2. Survey questions only for respondents without known diabetes

Diabetes risk factors and diabetes risk

Components of the German diabetes risk score (GDRS) [3, 4]

1) Are you physically active at least 5 hours a week? Including e. g., sport, gardening and cycling.

Possible answers: yes, no

2) Have you ever been told by a doctor that you have high blood pressure?

Possible answers: yes, no

3) Do you smoke?

Possible answers: yes, no

4) Have you smoked before?

Possible answers: yes, no

5) Have your biological parents been diagnosed with diabetes?

Possible answers: no, yes – one parent has diabetes, yes – both parents have diabetes, I don't know

6) Have your biological siblings been diagnosed with diabetes?

Possible answers: no – I don't have any siblings, no – my siblings were not diagnosed with diabetes, yes – at least one of my siblings has diabetes, I don't know

Other chronic diseases apart from diabetes

Do you have a chronic disease or a prolonged health problem? This refers to diseases or health problems that last or are expected to last for at least six months.

Possible answers: yes, no

(item of the Minimum European Health Module; MEHM [2])

Perceived diabetes risk

What do you think is your risk for getting diabetes over the next 5 years? (only respondents without known diabetes)

Possible answers: almost no risk, a low risk, a moderate risk, a high risk

(adopted from Kim at el. [5])

Health advice obtained by physician

1) Have you been at a medical practice or outpatient clinic in the last 12 months?

Possible answers: yes, no

Respondents who were at a medical practice or outpatient clinic in the last 12 months

2) During one of these visits in the last 12 months, have you received advise on the following health behaviors?
- Nutrition
- Body weight
- Physical activity
- Smoking
- Alcohol consumption
- Stress management
- Any other health behavior

Possible answers: yes, no (multiple answers were possible)

(items adopted from DEGS1; German Health Interview and Examination Survey for Adults [6])

Diabetes risk communicated by physician

Have you ever been told by a doctor that you have an increased diabetes risk?

Possible answers: yes, no

(self-developed)

3. Survey questions only for respondents with diabetes

Disease characteristics

1) When was your diabetes first diagnosed?

   Respondents reported age or time of diagnosis

2) Have you ever had or do you have one of the following sequelae or comorbidities?
- Diabetes-related kidney disease
- Diabetes-related eye disease
- Diabetes related nervous affection
- Diabetic foot lesions
- Amputations

Possible answers: yes, no (multiple answers were possible)

3) Have you ever been diagnosed with one of the following diseases or afflictions by a doctor besides your diabetes?
- Myocardial infarction
- Stroke
- Coronary heart disease

Possible answers: yes, no (multiple answers were possible)

(items adopted from DEGS1; German Health Interview and Examination Survey for Adults [6])

4) How is your diabetes currently treated?

- Insulin
- Other glucose lowering medications that are injected? (Other than insulin.)
- Glucose lowering oral medication
- Healthy diet
- Physical activity

(adopted from German Health Update survey [7])

Possible answers: yes, no (multiple answers were possible)

5) Regarding your method of blood sugar measurement, do you use one of the following?
- A blood glucose meter requiring a blood sample, i.e., pricking the tip of the finger or ear lobe
- A glucose sensor in the subcutaneous fatty tissue, i.e., continuous glucose monitoring systems and flash glucose monitoring systems

Possible answers: yes, no (multiple answers were possible)

(self-developed)

Personal control over diabetes

1) There is a lot which I can do to control my symptoms.
2) What I do can determine whether my diabetes gets better or worse.
3) The course of my diabetes depends on me.
4) I have the power to influence my diabetes.

Possible answers: strongly agree, agree, neither agree nor disagree, disagree, strongly disagree

(IPQ-R, personal control subscale [8, 9])

Health advice obtained by physician

In the last 12 months, have you received advise on the following health behaviors by your treatment team?

- Nutrition
- Body weight
- Physical activity
- Smoking
- Alcohol consumption
- Stress management
- Any other health behavior

Possible answers: yes, no (multiple answers were possible)

(items adopted from DEGS1; German Health Interview and Examination Survey for Adults [6])

References

1. Ernsting C, Dombrowski SU, Oedekoven M, Sullivan JL, Kanzler M, Kuhlmey A, et al. Using smartphones and health apps to change and manage health behaviors: a population-based survey. J Med Internet Res 2017 Apr 5;19(4):e101 [FREE Full text] [doi: 10.2196/jmir.6838] [Medline: 28381394]
2. Cox B, van Oyen H, Cambois E, Jagger C, le Roy S, Robine J, et al. The reliability of the Minimum European Health Module. Int J Public Health 2009;54(2):55-60. [doi: 10.1007/s00038-009-7104-y] [Medline: 19183846]
3. Mühlenbruch K, Joost HG, Boeing H, Schulze MB. Risk prediction for type 2 diabetes in the German population with the updated German Diabetes Risk Score (GDRS). Ernährungs Umschau 2014;61(6):90-93 [FREE Full text] [doi:10.4455/eu.2014.018]
4. Paprott R, Mühlenbruch K, Mensink GB, Thiele S, Schulze MB, Scheidt-Nave C, et al. Validation of the German Diabetes Risk Score among the general adult population: findings from the German Health Interview and Examination Surveys. BMJ Open Diabetes Res Care 2016;4(1):e000280 [doi: 10.1136/bmjdrc-2016-000280] [Medline: 27933187]
5. Kim C, McEwen LN, Piette JD, Goewey J, Ferrara A, Walker EA. Risk perception for diabetes among women with histories of gestational diabetes mellitus. Diabetes Care 2007 Sep;30(9):2281-2286. [doi: 10.2337/dc07-0618] [Medline: 17575087]
6. Scheidt-Nave C, Kamtsiuris P, Gößwald A, Hölling H, Lange M, Busch MA, et al. German health interview and examination survey for adults (DEGS) - design, objectives and implementation of the first data collection wave. BMC Public Health 2012 Sep 1;12:730 [FREE Full text] [doi: 10.1186/1471-2458-12-730] [Medline: 22938722]
7. Robert Koch-Institut. Fragebogen zur Studie 'Gesundheit in Deutschland aktuell': GEDA 2014/2015-EHIS [Questionnaire for the study 'German Health Update': GEDA 2014/2015-EHIS]. J Health Monitor 2017;2(1):105-135. [doi: 10.17886/RKI-GBE-2017-014]
8. Moss-Morris R, Weinman J, Petrie K, Horne R, Cameron L, Buick D. The Revised Illness Perception Questionnaire (IPQ-R). Psychol Health 2002;17(1):1-16. [doi: 10.1080/08870440290001494]
9. The Illness Perception Questionnaire. Krankheitsannahmen (The Illness Perception Questionnaire - R) [Disease Assumptions] URL: https://www.uib.no/ipq/pdf/IPQ-R-German.pdf [accessed 2019-04-10] [WebCite Cache ID 77Wm5u5KD]
