# Supplementary material for: Health App Use and Its Correlates Among Individuals With and Without Type 2 Diabetes: Nationwide Population-Based Survey
Source: JMIR Diabetes. 2020 May 20;5(2):e14396. doi: 10.2196/14396 (PMC7270854; doi:10.2196/14396)
Supplement: Multimedia Appendix 2 [file diabetes_v5i2e14396_app2.pdf]

Table 1. Sample characteristics of smartphone owners and nonsmartphone owners resulting from the initial sample of individuals without known diabetes n=2327. (The data are given as weighted percentage or mean [SD]. Missing data were less than 5% for all variables).

Variables	Smartphone owners n=1690[a] (74.72%)	Nonsmartphone owners n=637[a] (25.28%)
**Sociodemographic factors**		
Age in years, mean (SD)	43.69 (15.73)	67.14 (14.80)
Age in years (%)		
≥65	9.79	60.34
45-64	37.76	33.46
18-44	52.45	6.21
Gender, female (%)	50.78	54.33
Educational level (%)		
Low	23.61	51.65
Middle	45.48	32.36
High	30.63	15.99
**Diabetes-related risk factors**		
BMI (kg/m^2), mean (SD)	25.38 (4.50)	26.29 (4.73)
BMI (kg/m^2; %)		
BMI<25	53.31	42.37
25≤BMI<30	29.59	37.21
BMI≥30	15.07	18.15
Hypertension diagnosis (%)	25.82	52.61
Physical activity≥5 hours/week (%)	73.46	69.62
Smoking (%)		
Not smoking	45.13	56.14
Former smoking	24.29	25.84
Currently smoking	30.58	18.02
Family history of diabetes (%)[b]	21.34	25.08
**Psychological and health-related factors**		
Perceived health (%)		
Very good	28.30	15.08
Good	50.25	44.40
Moderate/poor/very poor	21.44	40.36
Chronic diseases (%)[c]	40.65	53.35
Perceived risk of getting diabetes (%)		
Almost no risk	41.37	43.87
Slight risk	40.91	35.00
Moderate risk	11.88	10.48

High risk	2.52	1.53
**Physician-provided information**		
Health advice obtained by physician (%)	48.30	42.18
Diabetes risk communicated by physician (%)	5.89	6.68

[a]Sample sizes (n) are given as unweighted.

[b]At least one parent or sibling was diagnosed with diabetes.

[c]Any chronic disease besides diabetes.

Table 2. Associations with smartphone ownership among people without known diabetes (n=2327). Analyses were based on imputed and weighted data. Model statistics: $\chi^2(20)=830.7$, $p<.001$. $R^2=.44$ (Nagelkerke); Hosmer & Lemeshow test: $\chi^2(8)=2.2$, $p=.19$ (values were averaged based on 20 imputed data sets).

Factors	Fully adjusted model		
	Odds ratio	95% CI	*P* value
**Sociodemographic factors**			
Age (years)[a]			
≥65	—[b]	—	—
45-64	5.33	4.07-6.98	< .001
18-44	37.37	24.77-56.40	< .001
Gender (reference: male)	1.17	0.92-1.49	
Educational level			
Low	—	—	—
Middle	1.62	1.24-2.12	< .001
High	2.42	1.75-3.34	< .001
**Diabetes-related risk factors**			
BMI (kg/m^2)			
BMI<25	—	—	—
25≤BMI<30	0.92	0.70-1.20	.53
BMI≥30	1.30	0.91-1.87	.15
Hypertension diagnosis (reference: no)	0.74	0.57-0.96	.02
Physical activity (≥5 hours per week; reference: <5 hours)	1.33	1.02-1.75	.04
Smoking			
Not smoking	—	—	—
Former smoking	1.41	1.06-1.87	.02
Currently smoking	1.44	1.07-1.95	.02
Family history of diabetes[c] (reference: no)	0.86	0.65-1.13	.28

**Psychological and health-related factors**			
Perceived health			
Moderate/poor/very poor	—	—	—
Good	1.72	1.28-2.33	< .001
Very good	1.58	1.05-2.38	.03
Chronic diseases[d] (reference: no)	1.06	0.81-1.40	.66
Perceived risk of getting diabetes			
Almost no risk	—	—	—
Slight risk	1.21	0.91-1.63	.19
Moderate risk	1.31	0.85-2.02	.22
High risk	1.48	0.58-3.75	.41
**Physician-provided information**			
Health advice obtained by physician (reference: no)	1.53	1.19-1.96	.001
Diabetes risk communicated by physician (reference: no)	0.88	0.53-1.44	.60

[a]Age categories were aligned with categories used in analyses of app use.

[b]Reference group.

[c]At least one parent or sibling was diagnosed with diabetes.

[d]Any chronic disease besides diabetes.

Table 3. Sample characteristics of smartphone owners and nonsmartphone owners resulting from the initial sample of individuals with known type 2 diabetes n=1149. (The data are given as weighted percentage or mean [SD]. Missing data were less than 5% for all variables).

Variables	Smartphone owners	Nonsmartphone owners
	n=481[a,b] (42.26%)	n=667[a,b] (57.64%)
**Sociodemographic factors**		
Age in years, mean (SD)	61.64 (11.73)	71.61 (10.44)
Age in years (%)		
≥65	41.9	22.5
18-64	58.1	77.5
Gender, female (%)	43.6	56.4
Educational level (%)		
Low	37.2	58.0
Middle	46.3	32.6
High	16.5	9.3

**Diabetes-related indicators**		
Diabetes duration in years, mean (SD)	11.79 (8.61)	15.24 (10.28)
BMI (kg/m²), mean (SD)	30.81 (6.11)	30.32 (5.52)
BMI (kg/m²; %)		
BMI<25	15.5	15.1
25≤BMI<30	33.3	37.2
BMI≥30	50.2	44.4
Diabetes-related complications[c], at least one (%)	32.2	35.7
Comorbidities[d], at least one (%)	24.4	35.7
Treatment with insulin (%)	41.0	48.9
Treatment with tablets (%)	75.0	65.9
Treatment with healthy diet or physical activity (%)	78.9	69.1
Method of blood sugar measurement[e] (%)		
Glucose meter with blood sampling	61.4	62.8
Subcutaneous fatty tissue in addition to or instead of a glucose meter	5.1	4.9
No use of measurements or no blood sugar measuring in the last 7 days	33.0	30.7
**Psychological and health-related factors**		
Perceived health (%)		
Very good	8.8	4.8
Good	43.3	39.0
Moderate/poor/very poor	47.8	56.0
Personal control over diabetes, mean (SD)[f]	16.86 (2.67)	15.37 (2.71)
**Physician-provided information**		
Health advice obtained by physician (%)	83.6	80.6

[a]Sample sizes (n) are given as unweighted.

[b]One case was excluded from analyses because he answered the question of owning a smartphone with “don’t know”.

[c]Including kidney disease, eye disease, nervous disease, diabetic foot lesions and amputations.

[d]Including heart attack, stroke and coronary heart disease.

[e]Multiple answers were eligible.

[f]Possible score range: 4-20.

Table 4. Associations with smartphone ownership among people with type 2 diabetes (n=1148). Analyses based on imputed and weighted data. Model statistics: $\chi^2(18)=256.9$, $p<.001$. $R^2=.28$ (Nagelkerke); Hosmer & Lemeshow test: $\chi^2(8)=4.7$, $p=.74$ (values were averaged based on 20 imputed data sets). One case was excluded from analyses because he answered the question of owning a smartphone with "don't know".

Factors	Fully adjusted model		
	Odds ratio	95% CI	*P* value
**Sociodemographic factors**			
Age (years)[a]			
≥65	—[b]	—	—
18-64	3.72	2.75-5.04	< .001
Gender (reference: male)	0.64	0.48-0.85	.002
Educational level			
Low	—	—	—
Middle	1.67	1.57-3.75	< .001
High	2.43	0.63-2.01	.75
**Diabetes-related indicators**			
Diabetes duration (years)	0.98	0.96-0.99	.004
BMI (kg/m^2)			
BMI<25	—	—	—
25≤BMI<30	0.83	0.55-1.25	.37
BMI≥30	0.93	0.62-1.41	.74
Diabetes-related complications[c]			
At least one complication (reference: no complication)	1.22	0.88-1.68	.24
Comorbidities[d]			
At least one comorbidity (reference: no comorbidity)	0.80	0.58-1.10	.16
Treatment with insulin (reference: no)	0.92	0.79-1.56	.55
Treatment with tablets (reference: no)	1.11	0.67-1.79	.71
Treatment with healthy diet or physical activity	1.37	0.99-1.91	.06
Method of blood sugar measurement			
Glucose meter with blood sampling	—	—	—
Blood glucose sensor in subcutaneous fatty tissue in addition to or instead of a glucose meter	1.15	0.61-2.19	.67
No use of measurements or no blood sugar measuring in the last 7 days	0.89	0.63-1.25	.49
**Psychological and health-related factors**			
Perceived health			

Moderate/poor/very poor	—	—	—
Good	1.41	1.04-1.91	.03
Very good	1.84	1.03-3.26	.04
Personal control over diabetes	1.16	1.10-1.23	< .001
**Physician-provided information**			
Health advice obtained by physician (reference: no)	0.72	0.49-1.05	.08

[c]Age categories were aligned with categories used in analyses of app use.

[b]Reference group.

[c]Complications asked in this survey were kidney disease, eye disease, nervous disease, diabetic foot lesions and amputations.

[d]Comorbidities asked in this survey were heart attack, stroke, and coronary heart disease.
