# Supplementary material for: Health App Use and Its Correlates Among Individuals With and Without Type 2 Diabetes: Nationwide Population-Based Survey
Source: JMIR Diabetes. 2020 May 20;5(2):e14396. doi: 10.2196/14396 (PMC7270854; doi:10.2196/14396)
Supplement: Multimedia Appendix 3 [file diabetes_v5i2e14396_app3.pdf]

Table 1. Number of missing values for the total sample of individuals without known diabetes and of individuals with type 2 diabetes.

Variables	Number of missing values, n[a] (%)[b]	
	Individuals without diabetes (n=2327)	Individuals with type 2 diabetes (n=1149)
**Smartphone ownership**	0 (0.00)	1 (0.09)
**Using a smartphone or app to improve one of the following behaviors:**		
quit smoking	1 (0.04)	3 (0.26)
be regularly physically active	1 (0.04)	0 (0.00)
maintain a healthy diet	0 (0.00)	1 (0.09)
reduce weight	0 (0.00)	0 (0.00)
take medication regularly	3 (0.13)	1 (0.09)
regulate blood pressure	0 (0.00)	0 (0.00)
regulate blood sugar	-[c]	0 (0.00)
No app is used to improve behaviors	2 (0.09)	0 (0.00)
**Sociodemographic factors**		
Age	0 (0.00)	0 (0.00)
Gender	0 (0.00)	0 (0.00)
Educational level	4 (0.17)	1 (0.09)
**Diabetes-related risk factors/ diabetes-related indicators**		
BMI (kg/m^2)	36 (1.55)	17 (1.48)
Hypertension diagnosis	1 (0.04)	-
Physical activity ≥5 hours/week	1 (0.04)	-
Smoking	0 (0.00)	-
Diabetes diagnosis in one or both biological parents	0 (0.00)	-
Diabetes diagnosis in at least one biological sibling	0 (0.00)	-
Diabetes duration	-	2 (0.17)
Diabetes-related complications		
Kidney disease	-	21 (1.83)
Eye disease	-	45 (3.92)
Nervous disease	-	19 (1.65)
Diabetic foot lesions	-	9 (0.78)
Amputations	-	2 (0.17)
Comorbidities		
Heart attack	-	2 (0.17)
Stroke	-	5 (0.44)
Coronary heart disease	-	11 (0.96)
Current treatment		
With insulin	-	1 (0.09)
Glucose lowering oral medication	-	0 (0.00)
Healthy diet	-	3 (0.26)
Physical activity	-	0 (0.00)
Method of blood measurement		

Blood glucose meter	-	15 (1.31)
Glucose sensor in the subcutaneous fatty tissue	-	0 (0.00)
**Psychological and health-related factors**		
Perceived health	1 (0.04)	2 (0.17)
Chronic diseases	1 (0.04)	-
Perceived risk of getting diabetes	90 (3.87)	-
Personal control over diabetes		
There is a lot which I can do to control my symptoms	-	24 (2.09)
What I do can determine whether my diabetes gets better or worse	-	30 (2.61)
The course of my diabetes depends on me	-	21 (1.83)
I have the power to influence my diabetes	-	8 (0.70)
**Health care factors**		
Health advice obtained by a physician on the following health behaviors in the last 12 months:		
nutrition	4 (0.17)	1 (0.09)
body weight	2 (0.09)	1 (0.09)
physical activity	2 (0.09)	8 (0.70)
smoking	2 (0.09)	9 (0.78)
alcohol consumption	2 (0.09)	6 (0.52)
stress management	1 (0.04)	5 (0.44)
any other health behavior	5 (0.22)	7 (0.61)
Diabetes risk communicated by physician	5 (0.22)	-

[a]Data are given as unweighted values (n).

[b]Data are given as weighted percentage.

[c]The variable was not assessed in this sample.
